# Abnormal splicing switch of *DMD's* penultimate exon compromises muscle fibre maintenance in myotonic dystrophy

**DOI:** 10.1038/ncomms8205

**Published:** 2015-05-28

**Authors:** Frédérique Rau, Jeanne Lainé, Laetitita Ramanoudjame, Arnaud Ferry, Ludovic Arandel, Olivier Delalande, Arnaud Jollet, Florent Dingli, Kuang-Yung Lee, Cécile Peccate, Stéphanie Lorain, Edor Kabashi, Takis Athanasopoulos, Taeyoung Koo, Damarys Loew, Maurice S. Swanson, Elisabeth Le Rumeur, George Dickson, Valérie Allamand, Joëlle Marie, Denis Furling

**Affiliations:** 1 Sorbonne Universités, UPMC Univ Paris 06, INSERM UMRS974, CNRS FRE3617, Center for Research in Myology, Institut de Myologie, GH Pitié-Salpêtrière, F-75013 Paris, France; 2 Sorbonne Universités, UPMC Paris 06, Département de Physiologie, Site Pitié-Salpêtrière, F-75013 Paris, France; 3 Université de Rennes 1, Institut de Génétique et Développement de Rennes, F-35043 Rennes, France; 4 Institut Curie, Centre de Recherche, Laboratoire de Spectrométrie de Masse Protéomique, F-75005 Paris, France; 5 Department of Molecular Genetics and Microbiology, Center for NeuroGenetics and the Genetics Institute, University of Florida, College of Medicine, Gainesville, Florida 32610, USA; 6 Department of Neurology, Chang Gung Memorial Hospital, Keelung 204, Taiwan; 7 Sorbonne Université, UPMC Univ Paris 06, UM 75, INSERM U1127, CNRS UMR7225, ICM, Paris, F-75013 Paris, France; 8 School of Biological Sciences, Royal Holloway—University of London, Egham, Surrey, TW20 0EX, UK

## Abstract

Myotonic Dystrophy type 1 (DM1) is a dominant neuromuscular disease caused by nuclear-retained RNAs containing expanded CUG repeats. These toxic RNAs alter the activities of RNA splicing factors resulting in alternative splicing misregulation and muscular dysfunction. Here we show that the abnormal splicing of *DMD* exon 78 found in dystrophic muscles of DM1 patients is due to the functional loss of MBNL1 and leads to the re-expression of an embryonic dystrophin in place of the adult isoform. Forced expression of embryonic dystrophin in zebrafish using an exon-skipping approach severely impairs the mobility and muscle architecture. Moreover, reproducing *Dmd* exon 78 missplicing switch in mice induces muscle fibre remodelling and ultrastructural abnormalities including ringed fibres, sarcoplasmic masses or Z-band disorganization, which are characteristic features of dystrophic DM1 skeletal muscles. Thus, we propose that splicing misregulation of *DMD* exon 78 compromises muscle fibre maintenance and contributes to the progressive dystrophic process in DM1.

## Abstract (web summary)

The splicing of the penultimate exon of the dystrophin gene is developmentally regulated. Here the authors show that the dysregulation of this exon's splicing leads to the expression of an embryonic dystrophin form with a C-terminus distinct from the adult isoform, which leads to muscle wasting in zebrafish and mice.

## Graphical abstract

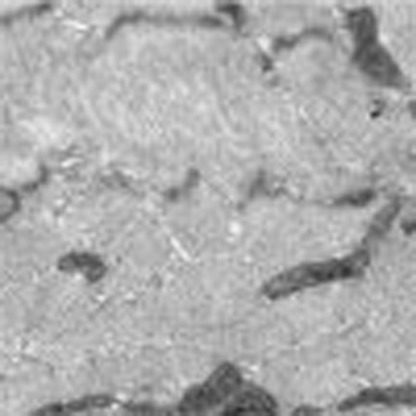

Myotonic Dystrophy type 1 (DM1), one of the most common neuromuscular disorders in adults, is characterized at the skeletal muscle level by progressive weakness, wasting and myotonia. DM1 is an autosomal dominant disorder caused by an expanded CTG repeat in the 3′-untranslated region of the *DMPK* gene^1^,^2^,^3^, in which the expression of pathogenic RNA leads to muscular dysfunction. It has been shown that CUG-expanded RNAs (CUGexp-RNAs) are retained in nuclear aggregates and alter the activities of Muscleblind-like (MBNL) and CELF1 RNA-binding factors involved in the regulation of alternative splicing during development^4^,^5^,^6^,^7^,^8^,^9^,^10^. Notably, functional loss of MBNL proteins due to their sequestration by nuclear CUGexp-RNA results in the abnormal embryonic splicing pattern of a subset of pre-mRNAs in DM1. Among them, missplicing of *CLCN1*, *INR, PKM, CACNA1S* and *BIN1* pre-mRNAs have been associated with myotonia, insulin resistance, perturbed glucose metabolism and muscle weakness, respectively, all symptoms of DM1 (refs 11, 12, 13, 14, 15, 16). Additional splicing misregulation events have been described in skeletal muscles of DM1 patients; however, their consequences on muscle function remain largely unknown. For instance, abnormal splicing regulation of *DMD* exon 78 that leads to the re-expression of an embryonic dystrophin isoform and strongly correlates with muscle disease severity in DM1 patients^17^,^18^, has not been investigated yet. The *DMD* gene is composed of 79 exons encoding a 427-kDa subsarcolemmal dystrophin protein in skeletal muscle. Dystrophin is part of a large dystrophin-associated glycoprotein complex (DGC) that stabilizes the membrane of muscle fibres and provides a scaffold for force transmission during muscle contraction, as well as transduction of extracellular-mediated signals to the muscle cytoskeleton^19^,^20^. Moreover, muscle degeneration resulting from the expression of truncated dystrophin in Becker muscular dystrophy or its loss in Duchenne muscular dystrophy highlights the importance of this subsarcolemmal protein for muscle function^21^,^22^.

The switch from embryonic to adult isoforms of dystrophin during muscle development involves fine-tuning coordinated alternative splicing transitions of two regions of the gene. The first concerns exons 71–74 that are all in-frame and may each be excluded leading to shorter dystrophin isoforms in embryonic muscles^23^,^24^,^25^. This splicing switch is also altered in muscle samples of DM1 patients, although it does not perturb dystrophin activity since mice deleted for *Dmd* exons 71–74 do not exhibit skeletal muscle abnormalities^26^. The second developmental splicing switch concerns the penultimate exon 78 (of 32 bp) that modifies the C-terminal (C-ter) tail of dystrophin^24^,^25^,^26^,^27^. Exclusion of exon 78 from *DMD* transcripts changes the open-reading-frame (ORF) of the last exon 79. The new ORF has a more downstream stop codon, producing a dystrophin with a 31 amino acids (aa) tail instead of a shorter 13aa tail when exon 78 is included (Supplementary Fig. 1a).

In this work, we investigate the consequences of *DMD* exon 78 splicing misregulation on muscle function. We show that *DMD* exon 78 splicing is regulated by MBNL1 during skeletal muscle development and modifies dystrophin C-terminus structure leading to a β-sheet C-terminus in the adult isoform in place of an amphipathic α-helix C-terminus in the embryonic isoform. This developmental transition is required for muscle function since forced exclusion of *dmd* exon 78 using an exon-skipping approach in zebrafish severely impairs the mobility and muscle architecture. Moreover, the expression of micro-dystrophin constructs in dystrophin-deficient mice demonstrates that the presence of the amphipathic α-helix C-terminus is not able to improve muscle function in contrast to the β-sheet C-terminus. Finally, we show that forced *Dmd* exon 78 skipping and subsequent embryonic dystrophin re-expression in wild-type (WT) mice leads to muscle fibre remodelling and ultrastructural abnormalities. Similar changes have been described in affected muscles of DM1 patients suggesting that abnormal splicing of *DMD* exon 78 could contribute to the progressive dystrophic process in this disease.

## Results

### *DMD* exon 78 splicing changes dystrophin C-terminus structure

**Figure 1:**
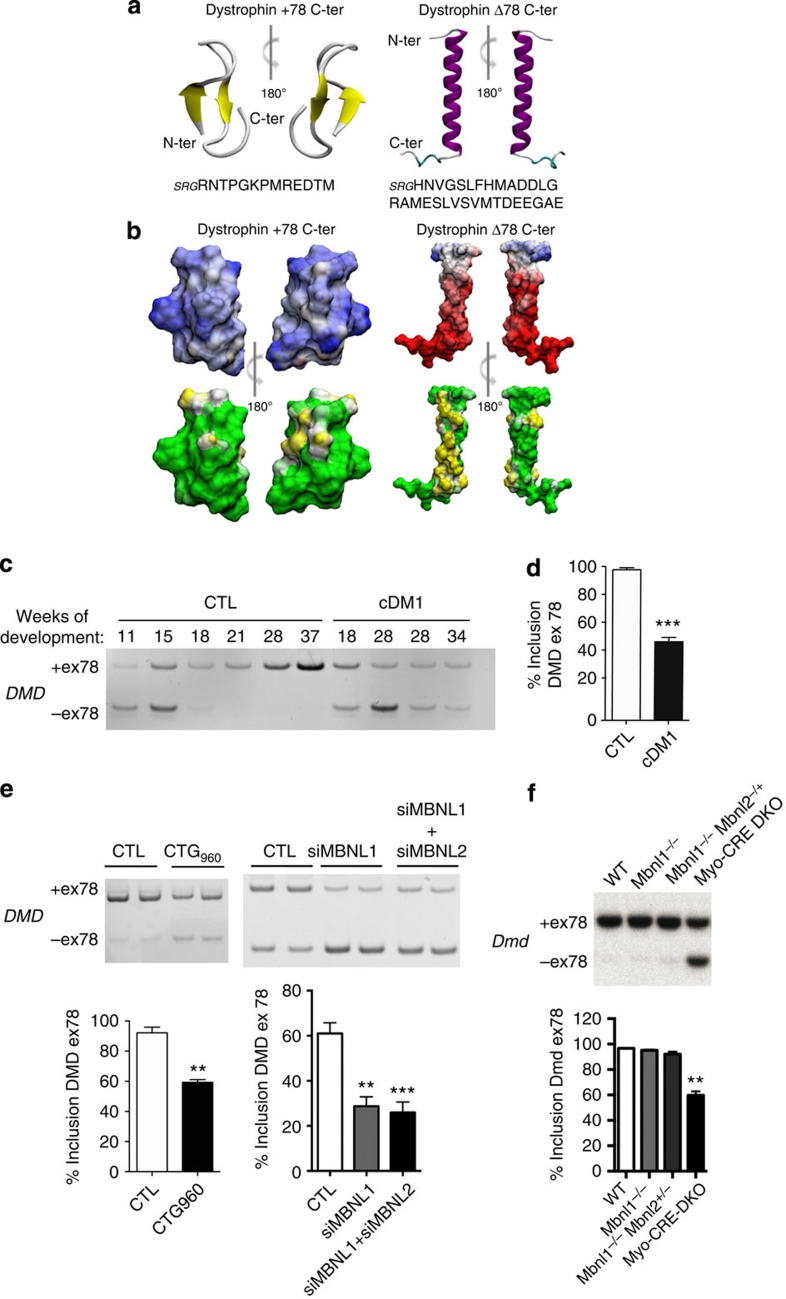
*DMD* exon 78 MBNL-regulated splicing switch changes dystrophin C-ter tail. (**a**) PEP-fold analysis of dystrophin +78 and dystrophin Δ78 C-ter structures. (**b**) Surface properties of PEP-fold models of dystrophin +78 and dystrophin Δ78 C-ter. Electrostatic potentials (upper panel) are shown in blue for electropositive and red for electronegative. Hydrophobic and hydrophilic surface potentials (lower panel) are coloured in yellow and green, respectively. (**c**) RT–PCR analysis of *DMD* exon 78 alternative splicing in human skeletal muscle samples from control (CTL) and congenital DM1 (cDM1) fetuses. (**d**) Quantification of *DMD* exon 78 inclusion in skeletal muscle samples from cDM1 compared with control fetuses aged between 20 and 37 weeks of development (*n*=3). (**e**) Upper left panel: RT–PCR analysis of endogenous *DMD* exon 78 inclusion in control differentiated human muscle cells with or without the expression of conditional 960 CTG repeats. Upper right panel: RT–PCR analysis of endogenous *DMD* exon 78 mRNA in control differentiated human muscle cells transfected with siRNAs against MBNL1 or both MBNL1 and MBNL2. Lower panel: quantification of *DMD* exon 78 inclusion (*n*=6 from three independent experiments). (**f**) RT–PCR analysis and quantification of endogenous *Dmd* mRNA in skeletal muscle samples from WT, *Mbnl1*^*−/−*^ Knockout, *Mbnl1*^*−/−*^*, Mbnl2*^*+/−*^ Knockout and myo-CRE muscle-specific *Mbnl1*^*−/−*^*, Mbnl2*^*−/−*^ double Knockout (myo-CRE DKO) (*n*=3). Bars indicates s.e.m. and ** indicates *P*<0.01; *** indicates *P*<0.001; Student's *t*-test).

To assess whether the splicing of *DMD* exon 78 affects the C-terminal structure of dystrophin, we modelled *in silico* the dystrophin C-ter tail primary sequence containing either 13aa (+78 C-ter) or 31aa (Δ78 C-ter). As illustrated in Fig. 1a, the modification of exon 79 ORF due to *DMD* exon 78 exclusion results in a complete reorganization of the dystrophin C-terminus molecular structure. The predicted fold of the +78 C-ter tail is a β-sheet with 16±1% of hydrophobic residues at the surface and a global positive charge (Fig. 1b and Supplementary Fig. 1b,c). In contrast, the Δ78 C-ter tail forms an amphipathic α-helix, containing 34±3% of hydrophobic residues and a global negative charge suggesting different biophysical and functional properties. It is noteworthy that the 31aa sequence corresponding to the embryonic dystrophin C-ter tail is highly conserved from worm to human supporting a critical function for this developmental-regulated domain^28^.

### Splicing switch of *DMD* exon 78 is regulated by MBNL1

In human, the splicing transition of *DMD* exon 78 occurs between 11 and 18 weeks of development, with its almost complete inclusion after 20 weeks of development, corresponding to the formation of the second generation of muscle fibres (Fig. 1c). In contrast this splicing switch is impaired after 20 weeks of development in fetal skeletal muscles of DM1 patients suffering from the severe congenital form. A significant 55% exclusion of *DMD* exon 78 was detected in skeletal muscles of congenital DM1 patients carrying large (>1,000) CTG expansions (Fig. 1d), as previously observed in affected muscles of adult DM1 patients^18^. To determine whether the pathogenic CTG expansion can interfere with the regulation of *DMD* exon 78 alternative splicing, we artificially expressed large expanded CUG repeats in differentiated control muscle cells^29^. We showed that the conditional expression of CUGexp-RNAs that form nuclear aggregates leads to the misregulation of *DMD* exon 78 alternative splicing (Fig. 1e, left panel). We then asked whether the MBNL splicing regulators, which are sequestered by CUGexp-RNA aggregates could regulate *DMD* exon 78 splicing. We focused on MBNL1 and MBNL2 knowing that MBNL1 is the major MBNL proteins expressed in adult skeletal muscle. We performed siRNA-mediated silencing of MBNL1 or both MBNL1 and MBNL2 in differentiated human muscle cells to mimic the functional loss of MBNL proteins in DM1 and showed that the silencing of MBNL1 is sufficient to promote an exclusion of *DMD* exon 78 (Fig. 1e, right panel). We further investigated the regulation of *Dmd* exon 78 in *Mbnl*-deficient mouse models. Alternative splicing of *Dmd* exon 78 was not altered in skeletal muscle of *Mbnl1*-deficient mice due to a functional compensatory elevation of Mbnl2 in this mouse model^30^. However, a significant exclusion of *Dmd* exon 78 in skeletal muscle of muscle-specific *Mbnl1*: *Mbnl2* double knockout (*Myo-Cre DKO*) mice (Fig. 1f) demonstrated that MBNL proteins regulate the developmental splicing switch of *DMD* exon 78. In addition, CELF1 that is upregulated in the presence of CUGexp-RNAs was excluded as a regulator because its overexpression in mice does not recapitulate *Dmd* exon 78 exclusion^31^. Altogether, these results indicate that *DMD* exon 78 missplicing in DM1 skeletal muscle is a direct consequence of MBNL1 loss-of-function caused by its sequestration in CUGexp-RNA aggregates.

### *Dmd* exon 78 exclusion impairs zebrafish muscle development

**Figure 2:**
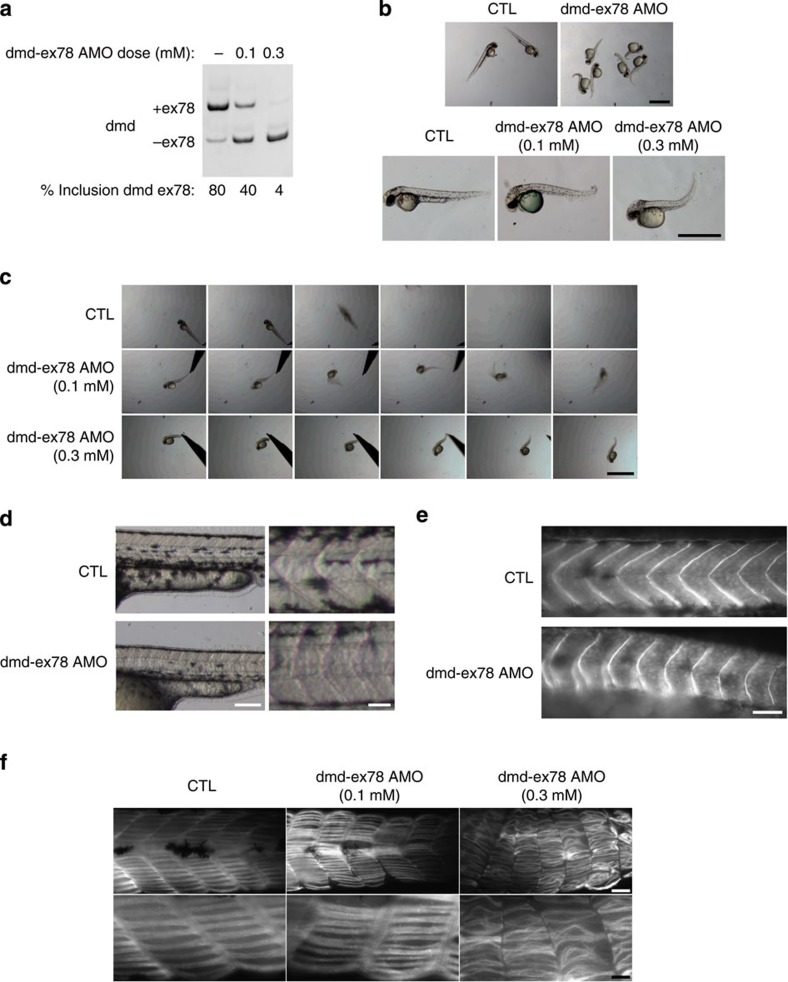
Exclusion of *dmd* exon 78 in zebrafish impairs skeletal muscle development. (**a**) RT–PCR of *dmd* exon 78 performed on total RNA extracts isolated from whole control and *dmd* Δ78 embryos (48 hpf). (**b**) Dose-dependant phenotype of *dmd* Δ78 embryos: control embryos (CTL) compared with moderate and severe affected *dmd* Δ78 morphants at 48 hpf (scale bar, 1 mm). (**c**) Touch-evoked escape test of control embryos compared with moderate and severe *dmd* Δ78 morphants (1 image/0.2 s; scale bar, 1 mm). (**d**) Abnormal myoseptum U-shape in *dmd* Δ78 morphants compared with V-shape in control embryos at 48 hpf (scale bar, 250 and 100 μm). (**e**) Dystrophin immunostaining (MANDRA1 antibody) of control embryos compared with *dmd* Δ78 morphants at 48 hpf. (**f**) Slow myosin immunostaining of control embryos compared with moderate and severe affected *dmd* Δ78 morphants at 48 hpf (scale bar, 50 and 10 μm).

To assess whether the splicing transition of *DMD* exon 78 is essential for muscle development, we developed an exon-skipping strategy to block the switch from the embryonic to the adult isoform without affecting the total amount of dystrophin. We used the zebrafish model because the 31aa C-ter tail is conserved in embryonic zebrafish dystrophin and exon 78 inclusion in zebrafish orthologous *dmd* transcripts also occurs during muscle formation, with a complete inclusion between 24 and 48 h post-fertilization (hpf)^27^,^32^. To prevent the developmental splicing switch of *dmd* exon 78, antisense morpholinos (AMO) complementary to the 3′ splicing site region were injected into one-cell-stage embryos to force the skipping of *dmd* exon 78, thereby maintaining the expression of embryonic dystrophin. A dose-dependent skipping of *dmd* exon 78 was measured in AMO-treated morphants (Fig. 2a) with no effect on total *dmd* mRNA level (*P*>0.05, *n*=3 independent pools of injected zebrafish, Student's *t*-test, Supplementary Fig. 2a). At the macroscopic level, abnormal splicing of *dmd* exon 78 in zebrafish led to major morphological abnormalities at 24 and 48 hpf. Caudal defects including shorter and twisted tails were observed in *dmd* Δ78 morphants and the severity of the phenotype (from moderate at low AMO concentration to severe at high AMO dose) correlated with the level of *dmd* exon 78 skipping (Fig. 2b). Touch-evoked escape tests revealed that *dmd* Δ78 morphants exhibited mobility impairments characterized by a disorganized movement pattern in moderately affected morphants to an abnormal ‘trembling' pattern in severely affected morphants (Fig. 2c and Supplementary Video). Regarding skeletal muscle organization, the shape of the myoseptum was altered, changing from the classic V-shape in WT embryos to a U-shape in *dmd* Δ78 morphants (Fig. 2d). Dystrophin immunostaining showed that dystrophin Δ78 was correctly localized at the myosepta and confirmed the abnormal ‘U' myoseptum shape in *dmd* Δ78 morphants (Fig. 2e). Immunostaining of slow myosin showed a disorganization of the muscle fibres in *dmd* Δ78 morphants with a marked perturbation of muscle fibre alignment and attachment/detachment to the myosepta in the severely affected morphants (Fig. 2f). These results are in agreement with the role of dystrophin in the formation of a stable muscle attachment to myosepta in zebrafish embryos^33^,^34^ and demonstrate that the developmental splicing transition of *dmd* exon 78 resulting in the removal of the 31aa C-ter tail of dystrophin is required to establish functional muscle structure during development. The 31aa C-ter tail renders the embryonic dystrophin unable to functionally replace adult dystrophin.

### The C-ter tail modulates dystrophin activity in muscle

**Figure 3:**
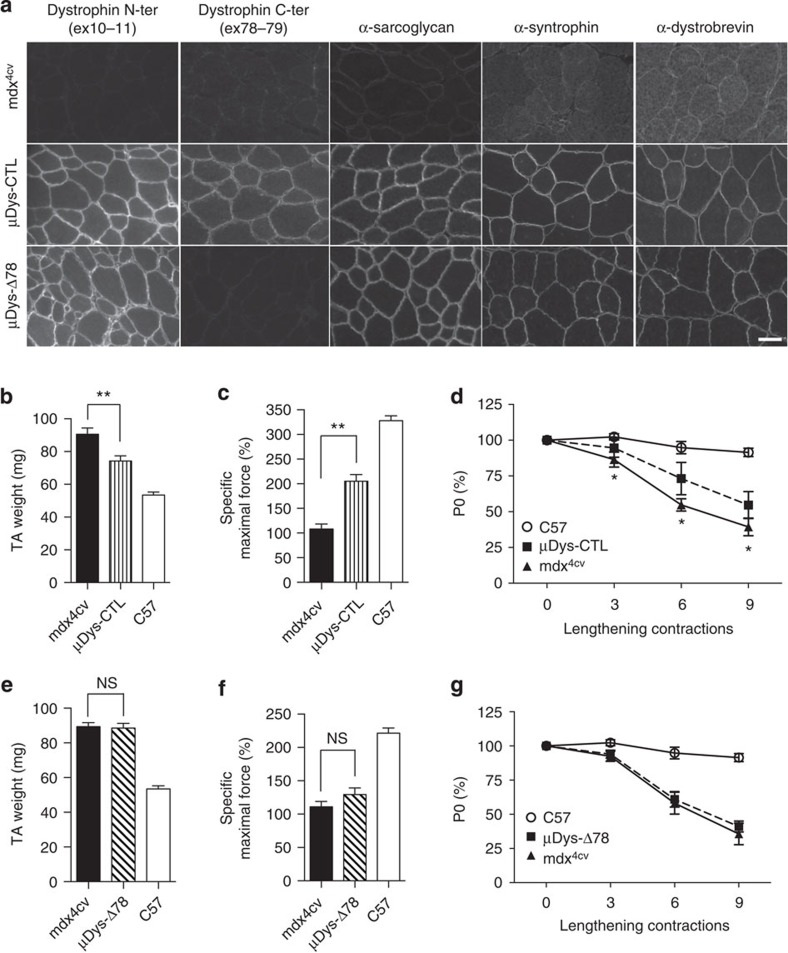
μ-dystrophin with a 31aa C-ter tail fails to ameliorate mdx muscle phenotype. (**a**) Dystrophin N-terminal domain (Manex1011B antibody), dystrophin C-terminus domain (Dys2 antibody), α-sarcoglycan, α-syntrophin and α-dystrobrevin immunostaining of *mdx*^4cv^ TA muscles injected with saline, AAV2/9-μDys-CTL or AAV2/9-μDys-Δex78 (scale bar, 50 μm). (**b,e**) TA muscle weight of C57BL/6 control mice (C57, *n*=8) compared with TA muscles of mdx^4cv^ mice (*n*=8) injected with saline (*mdx*^4cv^) or AAV2/9-μDys-CTL (**b**) or AAV2/9-μDys-Δex78 (**e**). (**c**,**f**) Specific maximal force (sP0) of TA muscles of C57BL/6 control mice (C57, *n*=8) compared with TA muscles of mdx^4cv^ mice (*n*=8) injected with saline (*mdx*^4cv^) or AAV2/9-μDys-CTL (**c**) or AAV2/9-μDys-Δex78 (**f**). (**d**,**g**) Resistance to eccentric contractions. Absolute maximal force (P0) following lengthening contractions of TA muscles of C57BL/6 control mice (C57, *n*=8) compared with TA muscles of mdx^4cv^ mice (*n*=8) injected with saline (*mdx*^4cv^) or AAV2/9-μDys-CTL (**d**) or AAV2/9-μDys-Δex78 (**g**). Bars indicate s.e.m. and ‘NS' indicates not significant; * indicates *P*<0.05; ** indicates *P*<0.01 compared to mdx^4cv^ condition; one-way analysis of variance with Tukey's multiple comparisons test.

To test whether the 31aa C-ter tail modulates dystrophin activity, we compared the efficiency of two micro-dystrophin (μDys) constructs containing either a 13aa tail (control micro-dystrophin μDys-CTL, due to the inclusion of exon 78) or a 31aa tail (μDys-Δ78 due to the lack of exon 78) in restoring muscle function of dystrophin-deficient (*mdx*^4cv^) mice. Adeno-associated virus (AAV2/9) vectors expressing μDys-CTL or μDys-Δ78 were injected locally in Tibialis Anterior (TA) muscles of *mdx*^4cv^ mice. Muscles transduced with each construct expressed similar levels of μDys transcripts (*P*>0.05, *n*=5, Student's *t*-test, Supplementary Fig. 2b,c) as well as comparable numbers of μDys-positive fibres (*P*>0.05, *n*=5, Student's *t*-test, Supplementary Fig. 2d) and showed the expected localization of μDys at the sarcolemma (Fig. 3a). Of note, the Dys2 antibody directed against a C-ter dystrophin epitope encoded by *DMD* exons 78–79 confirmed the modification of the μDys-Δ78 C-ter tail (Fig. 3a). As described by others for functional μDys constructs^35^,^36^,^37^,^38^,^39^,^40^,^41^, *mdx*^4cv^ muscles injected with μDys-CTL showed a significant reduction in TA muscle weight (Fig. 3b) and a significant improvement of the specific maximal force (Fig. 3c) when compared with the saline-injected contralateral muscles. We then determined the ability of μDys to protect skeletal muscle from injury by testing its resistance to eccentric contractions. A partial but significant improvement of resistance to eccentric contraction was observed in μDys-CTL injected muscles compared with saline-injected muscles (*P*<0.05; Fig. 3d). In contrast, *mdx*^4cv^ muscles injected with μDys-Δ78 did not exhibit a reduction in TA muscle weight when compared with saline-injected contralateral muscles (Fig. 3e). Moreover, the expression of μDys-Δ78 did not improve the specific maximal force (Fig. 3f) and muscle resistance to eccentric contractions in *mdx*^4cv^ mice (Fig. 3g). Because μDys-Δ78 was not able to ameliorate *mdx*^4cv^ muscle function, we evaluated whether the presence of a 31aa C-ter tail in the μDys-Δ78 compromised the recruitment of the DGC, which is not localized at the membrane in control *mdx*^4cv^ muscle fibres (Fig. 3a). We found that DGC partners such as α-sarcoglycan but also α-syntrophin and α-dystrobrevin that have binding sites localized near the C-ter domain encoded by exon 78 (refs 42, 43) are correctly localized at the sarcolemma in both μDys-CTL- and μDys-Δ78-injected muscles (Fig. 3a). However, in contrast to μDys-CTL, μDys-Δ78 does not improve *mdx*^4cv^ muscle function indicating that the 31aa C-ter tail perturbs the functions of dystrophin related to the protection of muscle performance from mechanical stresses induced by repeated contractions.

### DMD exon 78 is required for muscle fibre maintenance

**Figure 4:**
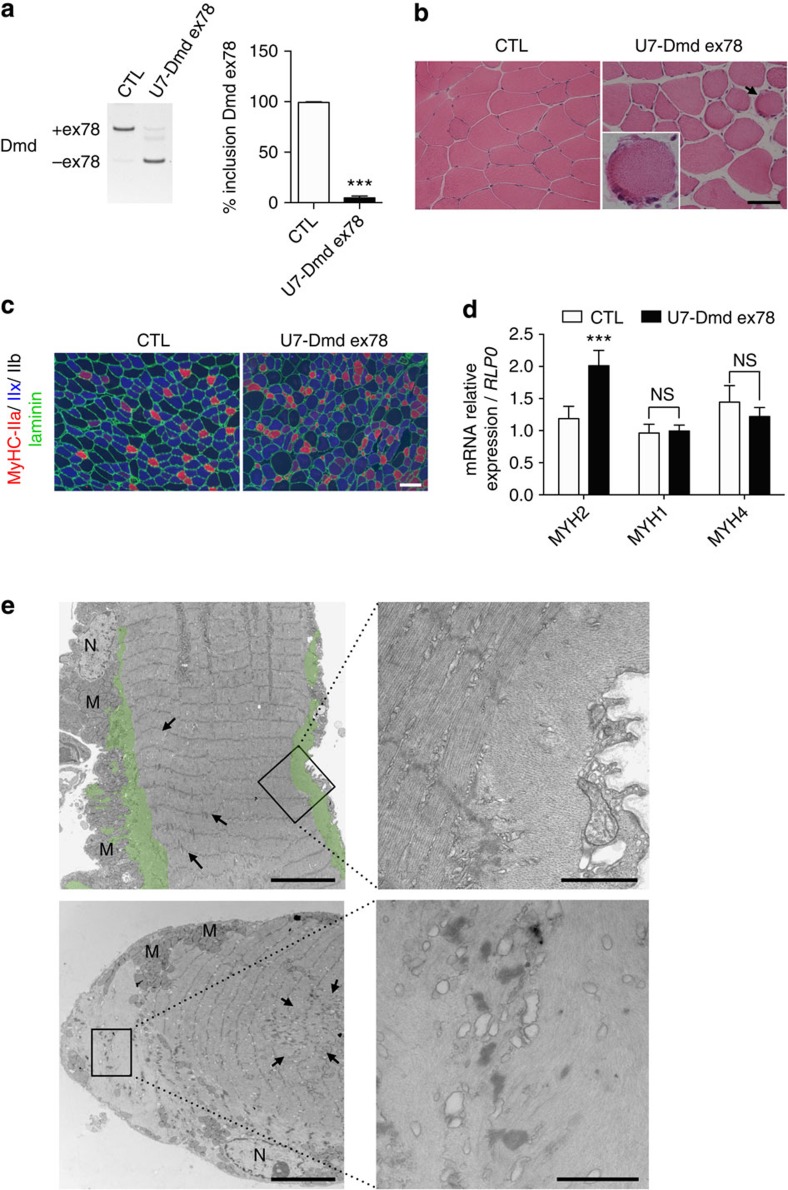
Dystrophin exon 78 is required for muscle structure maintenance. (**a**) RT–PCR analysis and quantification of *Dmd* exon 78 inclusion in TA muscles (*n*=10) injected with AAV-U7-*Dmd*-ex78 compared with contralateral TA muscles injected with saline (CTL). (**b**) Hematoxylin and eosin staining in TA muscles injected either with saline (CTL) or AAV-U7-*Dmd*-ex78 (scale bar, 50 μm). Inset: higher magnification (× 2.5) of a structure reminiscent of ringed fibre shown by the arrow. (**c**) Fibre types in TA muscles injected either with saline (CTL) or AAV-U7-*Dmd*-ex78 were determined by MyHC immunostaining: MyHC-IIa in red, MyHC-IIx in blue, MyHC-IIb dark, laminin in green. Purple fibres correspond to MyHC-IIa and MyHC-IIx positive fibres (scale bar, 100 μm). (**d**) Quantification of MyHC mRNA levels by quantitative RT–PCR (*n*=10). Bars indicate s.e.m. and ‘NS' indicates not significant; *** indicates *P*<0.001; Student's *t*-test). (**e**) Ultrastructure of representative, longitudinally cut, fibres in AAV-U7-*Dmd*-ex78-injected TA. Upper panel: ‘ringed fibre'. Sarcomeres are mainly longitudinally oriented, but just under the sarcolemma, a band of myofibrils (pseudo-coloured in green) is transversally oriented as evidenced in the enlarged zone (N, nucleus; M, mitochondria). Lower panel: sarcoplasmic mass. The sarcoplasm beneath its sarcolemma appears nearly devoid of myofilaments and the higher magnification shows some electrodense remnants of Z line material and vacuoles of swollen sarcoplasmic reticulum. In addition focal zones with Z line streaming are also observed in fibres of AAV-U7-*Dmd*-ex78-injected muscles (arrows in left panels). Left panels scale bars, 5 μm and rigth panels scale bars, 1 μm.

To further determine whether the re-expression of the endogenous embryonic dystrophin isoform having a 31aa C-ter tail instead of a 13aa tail is sufficient to affect muscle homeostasis, we artificially skipped *Dmd* exon 78 in skeletal muscles of adult WT mice using an antisense strategy. For this purpose, an engineered AAV2/9 vectors expressing optimized U7-snRNAs-antisense targeting *Dmd* exon 78 splicing sites were injected in TA muscles of adult WT mice. Reverse transcription PCR (RT–PCR) analysis demonstrated that the continuous expression of U7-*Dmd*-ex78 antisense induces a near-complete exclusion of *Dmd* exon 78 (Fig. 4a) without affecting the total *Dmd* mRNA level (*P*>0.05, *n*=10, paired *t*-test, Supplementary Fig. 3a). Mass-spectrometry analysis confirmed the switch of the dystrophin C-ter tail from 13aa to the expected 31aa in U7-*Dmd*-ex78 injected muscles (Supplementary Fig. 3b). As observed with the μDys-Δ78 construct, dystrophin Δ78 and DGC partners such as α-syntrophin and α-dystrobrevin were correctly localized at the sarcolemma (Supplementary Fig. 3c). Six months post-injection, evaluation of muscle contractile properties showed that U7-*Dmd*-ex78 injected muscles have a significant reduction of absolute maximal force (*P*<0.01, *n*=10, paired *t*-test; Supplementary Fig. 3d) that is related to a decrease in muscle weight (*P*<0.05 *n*=10, paired *t*-test; Supplementary Fig. 3e) since specific maximal force remains unchanged (*P*⩾0.05, *n*=10, paired *t*-test; Supplementary Fig. 3f) when compared with saline-injected contralateral muscles. Histological analysis of U7-*Dmd*-ex78 injected muscles revealed fibre size heterogeneity as well as structure reminiscent of ringed fibres (Fig. 4b) and a significant reduction of maximal muscle cross-section area (CSA) compared with the saline-injected contralateral muscles (*P*<0.01 *n*=10, paired *t*-test; Supplementary Fig. 3g). Muscle fibre composition of U7-*Dmd*-ex78 injected TA muscles was also altered with a reduced CSA of oxidative type 2a fibres, which is associated with an increase in their number and an increased CSA of glycolytic type 2b fibres (Fig. 4c). Quantitative RT–PCR analysis of myosin heavy chain (MyHC) mRNA levels confirmed the significant increase of the more oxidative type 2a fibres in the U7-*Dmd*-ex78 injected muscles (Fig. 4d). No centrally located nuclei were observed in U7-*Dmd*-ex78 injected muscles indicating that the associated muscle remodelling was not due to an active degeneration/regeneration process as observed in dystrophin-deficient muscles.

Next, we performed electron microscopy analyses of U7-*Dmd*-ex78 injected muscles to determine whether the modification of the dystrophin C-ter tail impacts the structure of the muscle fibre. Six months post injection, we observed that the 13aa C-ter tail replacement by the 31aa C-ter tail in dystrophin leads to distinct ultrastructural abnormalities: (i) myofibril disorganization showing myofilaments perpendicularly orientated at the periphery of, or across, oxidative fibres, which is characteristic of ringed fibres (Fig. 4e, upper panels and Supplementary Fig. 4, upper panels), (ii) sarcoplasmic masses displaying large areas of sarcoplasm at the fibre periphery with a complete absence of myofibrils but numerous vacuoles (Fig. 4e, lower panels), (iii) focal internal disorganization of the Z-band (Fig. 4e, arrows) (iv) and finally, dilated sarcoplasmic reticulum in glycolytic fibres (Supplementary Fig. 4, lower panels). These findings showed that the 31aa C-ter tail switch mediated by the exclusion of exon 78 in *Dmd* transcripts did not allow dystrophin to maintain correct myofibril architecture and ultrastructural organization of muscle fibres. Altogether these results indicate that the dystrophin function involved in the maintenance of muscle fibre organization during muscle contraction is impaired by the aberrant expression of the 31aa C-ter α-helix.

## Discussion

In this study we demonstrate that the developmental splicing transition of *DMD* exon 78, which is regulated by MBNL1 splicing factor, is essential for skeletal muscle development and muscle fibre organization. During muscle development, the sequence and structure of the dystrophin C-ter tail is switched from a 31aa residues amphipathic α-helix in the embryonic dystrophin isoform to a 13aa residues β-sheet fold in the adult dystrophin isoform. Developmental alternative splicing of the penultimate exon 78 is highly conserved throughout vertebrates and the 31aa C-ter tail of the embryonic dystrophin isoform is more conserved than the adult C-ter tail^44^ suggesting a critical role for this domain. Indeed, we show that the prolonged expression of the embryonic dystrophin isoform carrying a 31aa C-ter tail during zebrafish development, by forced *dmd* exon 78 skipping, leads to muscle architecture and mobility impairment. Of note, partial skipping of *dmd* exon 78 in zebrafish is sufficient to produce moderate defects. Whether it is the expression of the embryonic dystrophin isoform or the absence of the adult isoform that is more likely to cause this phenotype remains to be determined. Nevertheless, our results indicate that the 31aa C-ter α-helix impacts dystrophin activity. Indeed, the embryonic dystrophin isoform is not able to replace adult dystrophin function as shown by μDys experiments in dystrophin-deficient mice. Because the aberrant expression of dystrophin C-ter α-helix tail could have different consequences on skeletal muscle behaviour depending on whether it occurs during the developmental stages or the post-natal period, we mimicked *Dmd* exon 78 splicing misregulation in adult mouse muscles. Forced *Dmd* exon 78 skipping does not compromise membrane integrity or lead to continuous cycles of fibre degeneration/regeneration as observed in dystrophin-deficient muscles, but alters muscle fibre size and composition as well as its organization as shown by the formation of ringed fibres, sarcoplasmic masses or Z-band disorganization. This modification of the dystrophin C-ter tail does not affect dystrophin localization in the muscle fibre but prevents the protection of the sarcomeric apparatus from contraction-induced stress. Altogether, these results show that dystrophin function involved in the maintenance of muscle fibre organization during muscle contraction is impaired by *DMD* exon 78 missplicing, in particular the aberrant re-expression of the 31aa C-ter α-helix.

Misregulation of alternative splicing events in DM1 disease is caused by the expression of mutant RNA containing expanded CUG repeats that alter the activities of the splicing factors. Our results show that the functional loss of MBNL1 splicing regulator due to its sequestration in nuclear-retained CUGexp-RNAs is responsible for abnormal *DMD* exon 78 splicing in DM1 skeletal muscle cells. Among the altered splice events confirmed in skeletal muscles of DM1 patients, changes of *DMD* exon 78 splicing strongly correlated with disease severity^18^. Up to 50% of abnormal splicing switch of *DMD* exon 78 was reported in affected muscle biopsies of DM1 patients^17^,^18^, however, it is unclear whether the percentage of embryonic dystrophin re-expression varies among muscle fibres leading to higher level of splicing changes in certain fibres. Remarkably, ultrastructure abnormalities such as ringed fibres, sarcoplasmic masses or Z-band disorganization observed in muscle fibres of mice expressing skipped *Dmd* exon 78 isoform were also described in skeletal muscle biopsies of DM1 patients^45^,^46^,^47^. Although similar muscle fibre abnormalities have also been reported in other muscular dystrophies, these ultrastructure changes are commonly found and represent characteristic features of DM1 skeletal muscles. Moreover, adult mouse muscles re-expressing embryonic dystrophin isoform present a typical combination of fibre type changes that is also observed in affected skeletal muscle of DM1 patients: increased number of oxidative slow-twitch fibres with a selective atrophy of these fibres and a hypertrophy of the fast-twitch fibres. As abnormal splicing switch of *Dmd* exon 78 compromises muscle fibre maintenance in adult mouse muscle with phenotypic changes that correlate with abnormalities described in skeletal muscle biopsies of DM1 patients, we suggest that the inappropriate re-expression of the embryonic dystrophin isoform carrying a 31aa C-ter tail could contribute to the progressive muscle fibre deterioration in DM1.

In conclusion, our work underlines the critical MBNL1-dependent regulation of *DMD* exon 78 splicing for both skeletal muscle development and muscle fibre maintenance. It also proposes that the splicing misregulation of *DMD* exon 78 induced by the expression of expanded CTG repeats participates to the progressive dystrophy in DM1. These results are of utmost importance for our understanding of the characteristic dystrophic process in myotonic dystrophy but also in the context of therapeutic development of functional μDys or truncated dystrophins generated by exon-skipping technologies in Duchenne muscular dystrophy.

## Methods

### Molecular modelling

Molecular models were produced by using the *de novo* PEP-fold method^48^ through the dedicated webserver (http://bioserv.rpbs.univ-paris-diderot.fr/services/PEP-FOLD/), given no homology sequence were found for the exon 78 and exon 79 of dystrophin. Submitted primary sequences were SRGRNTPGKPMREDTM and SRGHNVGSLFHMADDLGRAMESLVSVMTDEEGAE, respectively, for the +78 C-ter (adult ORF) and Δ78 C-ter (fetal ORF). Secondary structure analyses from the PEP-Fold calculations are shown as diagrams indicating a probability (in %) to adopt a coil (green), sheet (blue) or helix (red) fold. Electrostatic potentials were computed by using the APBS program^49^ and hydrophobic potentials and molecular surfaces were provided by the Platinum webserver (http://model.nmr.ru/platinum/)^50^.

### Zebrafish embryos microinjection and whole-mount immunolabelling

Injections of zebrafish were performed in 1–4 cell stage blastulae. Antisense morpholino oligonucleotide (AMO; 5′- GTCCGCCTCCTTAGACAGAGGAAAA -3′) was design and manufactured by Gene Tools to bind and inhibit specifically *dmd* exon 78 inclusion. Dose-dependence curves of AMO were performed (0.1–0.7 mM) and AMOs were injected at a concentration of 0.1 and 0.3 mM to minimize morpholino-induced developmental delay and toxicity and to yield a consistent motor phenotype. After harvesting at 48 hpf developmental stages, zebrafish embryos were fixed with paraformaldehyde 4% for 2 h and dehydrated with MetOH 50% during 5 min followed by MetOH 100% for 5 min. After permeabilization with 70% EtOH, 20% acetic acid in PBS and blocking with 5% goat serum, 0.5% triton X-100 (Sigma-Aldrich), embryos were incubated overnight at 4 °C with mouse monoclonal anti-dystrophin (MANDRA1, Sigma 1:1,000) or mouse monoclonal F59 anti-slow-twitch myosin (1:10) in a 5% goat serum, 0.5% triton X-100 solution. Then the embryos were washed in PBS containing 0.1% Tween-20, incubated overnight at 4 °C with cy3-conjugated goat anti-mouse secondary antibody (Life Technologies; 1:400), washed with PBS containing 0.1% Tween-20 and mounted in glass slides.

### Touch-evoked escape response

Morphology and behavioural touch responses were assessed. Only fish with no obvious developmental deficits were selected to determine the touch-evoked escape response. For escape swimming at 48 hpf, embryos were touched lightly at the level of the tail with a pincer. Fish that were unable to escape were touched several times (3–4 times) to ascertain their failure to respond.

### Human muscle samples and muscle cells cultures

Skeletal muscle samples were obtained from autopsies, in accordance with the French legislation on ethical rules. Control and cDM1 muscle samples were from aborted fetuses showing, respectively, no sign of neuromuscular disease (control) and clinical symptoms of congenital DM1 form with large CTGn>1,000 repeats. Muscle cell cultures were derived from primary human satellite cells as previously described^51^. In brief, myoblasts were grown in DMEM/199 (ratio 4/1) medium (Gibco/Life Technologies) supplemented with 20% fetal bovine serum and antibiotics. For differentiation, growth medium was removed from confluent cultures and replaced by DMEM medium. All cultures were incubated at 37 °C in a humid atmosphere containing 5% CO_2_. For conditional expression of CTG repeats, myoblasts were transduced with lentiviral vectors expressing a 960 CTG Tet-on construct^29^. For *in vitro* MBNL silencing experiments, muscle cells were transfected with siRNAs (50 nM) directed against MBNL1 (5′- CAGACAGACUUGAGGUAUGdTdT -3′; Eurogentec) and/or MBNL2 (5′- GAAGAGUAAUUGCCUGCUUUUdTdT -3′; Eurogentec) using Lipofectamine RNAiMAX reagent (Life Technologies) according to the manufacturer's protocol.

### *In vivo* gene transfer

All the mouse procedures were done according to protocol approved by the Committee on Animal Resources at the Centre d' Experimentation Fonctionnelle of Pitie-Salpetriere animal facility and under appropriate biological containment. AAV2/9 vectors were produced using three-plasmid constructs protocol. For μDys expression, 2-month-old mdx^4CV^ mice were injected into the TA with 50 μl of AAV2/9-μDys vectors containing 2.5 × 10^9^ viral genomes (vg). The murine optimized μDys-CTL construct incorporates deletion of spectrin-like repeat domain 4–23 and the μDys Δ78 construct contains an additional deletion of exon 78. For optimized U7-snRNA-antisense expression, U7-Dmd-ex78 construct was done as previously described^12^ and adult *C57BL/6* WT mice were injected into the TA with 40 μl of AAV2/9-U7-Dmd-ex78 containing 1 × 10^11^ vg. For each mouse, the left TA muscle was injected with AAV and the contralateral muscle was injected with vehicle alone (PBS). Animals were sacrificed 2 months after AAV-μDys injection or 6 months after AAV-U7-Dmd-ex78 injection and muscles were collected, snap-frozen in liquid nitrogen-cooled isopentane and stored at −80 °C.

### RNA extraction and RT–PCR analysis

RNAs were isolated using Tri Reagent (Sigma) according to the manufacturer's protocol. 1 μg of RNA was reverse transcribed using M-MLV first-strand synthesis system according to the manufacturer's instructions (Invitrogen) in a total of 20 μl. One microlitre of cDNA preparation was subsequently used in a semi-quantitative PCR analysis according to standard protocol (ReddyMix, Thermo Scientific). PCR amplification was carried out for 20–35 cycles within the linear range of amplification for each gene. PCR products were resolved on 1% agarose or 5% non-denaturing polyacrylamide (for splicing) gels, BET stained and quantified with ImageJ software. The ratios of exon inclusion/exclusion were quantified as a percentage of inclusion/exclusion relative to total intensity of isoform signals. To quantify the mRNA expression, real-time PCR was performed using a Lightcycler 480 (Roche). Reactions were performed with SYBR Green kit (Roche) according to the manufacturer's instructions. PCR cycles were a 15-min denaturation step followed by 50 cycles with a 94 °C denaturation for 15 s, 58 °C annealing for 20 s and 72 °C extension for 20 s. Mouse *Rrlp0* mRNA or zebrafish elfa (elongation factor alpha) mRNA were used as standard. Data were analysed with the Lightcycler 480 analysis software. PCR primer sequences are listed in Supplementary Table S1.

### Immunohistochemistry and histology

Hematoxylin and eosin staining was used to examine the overall muscle morphology of 10 μm TA muscle sections. For immunohistochemistry, muscle cryo-sections were stained using Mouse on Mouse (M.O.M) kit (Vector Labs). Primary antibodies were incubated overnight at 4 °C followed by three washes with PBS-0.1% Tween and incubated with goat anti-mouse or goat anti-rabbit secondary antibodies (Life Technologies; 1:400) conjugated to Alexa 488, Alexa 555 or Alexa 647. Antibodies against dystrophin (Manex1011B, 1:100, mouse monoclonal, gift from Dr Glenn Morris; Dys1 and Dys2, 1:100, mouse monoclonal, Novocastra; MANDRA1, 1:1,000, Mouse monoclonal, Sigma-Aldrich), α-syntrophin (rabbit polyclonal, 1:200, Abcam), α-dystrobrevin (mouse monoclonal, 1:200, BD Biosciences); anti-MHCIIa (SC71, 1:3, mouse monoclonal IgG1; Hybridoma DSHB), anti-MHCIIX (6H1, 1:2, mouse monoclonal IgM; Hybridoma DHSB), laminin (1:300, rabbit polyclonal, Chemicon) were used.

### Electron microscopy

TA muscles were dissected, cut into small pieces and immediately fixed in 2% glutaraldehyde, 2% paraformaldehyde, 0.1 M phosphate buffer. After abundant washes and 2% OsO4 post-fixation samples were dehydrated at 4 °C in graded acetone including a 1% uranyl acetate in 70° acetone step and were finally embedded in Epon resin. Thin (70 nm) sections were stained with uranyl acetate and lead citrate, observed using a Philips CM120 electron microscope (Philips Electronics NV) and photographed with a digital SIS Morada camera.

### *In situ* force measurement

The isometric contractile properties of TA muscle were studied *in situ*. Mice were anaesthetized with pentobarbital (60 mg kg^−1^ intraperitoneally). The knee and foot were fixed with clamps and pins. The distal tendon of the TA muscle was attached to a lever arm of a servomotor system (305B, Dual-Mode Lever). Data were recorded and analysed on a microcomputer using PowerLab system (4SP, ADInstruments) and software (Chart 4, ADInstruments). The sciatic nerve (proximally crushed) was stimulated by a bipolar silver electrode using a supramaximal (10-V) square wave pulse of 0.1-ms duration. All contractions were made at an initial length L_0_ (length at which maximal tension was first obtained during tetanic contractions). Absolute maximal isometric tetanic force was measured during isometric contractions in response to electrical stimulation (frequency of 25–150 Hz, train of stimulation of 500 ms). Maximal specific isometric force was calculated by dividing absolute maximal isometric force by muscle weight.

Resistance to eccentric (lengthening) contractions of TA muscles was then evaluated by measuring the force drop following eccentric contractions. A maximal isomeric contraction of the TA muscle was initiated during the first 500 ms. Then, muscle lengthening (1.1 mm, 10% L_0_) at a velocity of 0.5 mm s^−1^ (∼0.5 L_0_ s^−1^) was imposed during the last 200 ms. Nine lengthening contractions of the TA muscles were performed, each separated by a 60-s rest period. Maximal isometric force was measured after each eccentric contraction and expressed as a percentage of the initial maximal isometric force.

### Dystrophin immunoprecipitation and LC-MS/MS analysis

TA muscles of mice were homogenized in ice-cold lysis buffer (150 mM NaCl, 1% Triton, 0.1% SDS, 1% sodium deoxycholate, 150 mM Tris-HCl, pH 8) containing Complete Protease Inhibitors and PhosSTOP cocktails (Roche Diagnostics). After 30-min incubation on ice, the samples were centrifuged at 14,000*g* for 15 min to pellet cell debris and protein concentration of the supernantant fraction was determinated by Pierce BCA Protein assay kit (Thermo Scientific). Immunprecipitation was performed using 1 mg of protein extract and Pierce Crosslink Immunprecipitation kit (Thermo Scientific) according to the manufacturer's instructions using 25 μg of MANEX1011B antibody. Proteins were eluted in Laemmli Reducing Sample Buffer and separated on SDS–polyacrylamide gel electrophoresis.

Excised gel slices were washed and proteins were reduced with 10 mM DTT before alkylation with 55 mM iodoacetamide. After washing and shrinking of the gel pieces with 100% acetonitrile, in-gel digestion was performed using trypsin (Sequencing Grade, Promega) overnight in 25 mM ammonium bicarbonate at 30 °C. The extracted peptides were analysed by nano-LC-MS/MS using an Ultimate 3,000 system (Dionex S.A.) coupled to an linear trap (LTQ)-Orbitrap XL mass spectrometer (Thermo Fisher Scientific, Bremen, Germany). Samples were loaded on a C18 precolumn (300 μm inner diameter × 5 mm; Dionex) at 20 μl min^−1^ in 5% acetonitrile, 0.1% TFA. After 3 min of desalting, the precolumn was switched on line with the analytical C18 column (75 μm inner diameter × 50 cm; C18 PepMap, Dionex) equilibrated in solvent A (2% acetonitrile, 0.1% formic acid). Bound peptides were eluted using a two-step linear gradient of 157 min (from 0 to 30% (v/v)) and 20 min (from 30 to 50%) of solvent B (80% acetonitrile, 0.085% formic acid) at a 150 nl min^−1^ flow rate and an oven temperature of 40 °C. Data-dependent acquisition was performed on the LTQ-Orbitrap mass spectrometer in the positive ion mode. Survey MS scans were acquired in the Orbitrap on the 350–1,000 *m/z* range with the resolution set to a value of 100,000. Each scan was recalibrated in real time by co-injecting an internal standard from ambient air into the C-trap (‘lock mass option'). The five most intense ions per survey scan were selected for collision-induced dissociation fragmentation and the resulting fragments were analysed in the LTQ. Target ions already selected for MS/MS were dynamically excluded for 30 s.

Data were acquired using the Xcalibur software (version 2.2) and the resulting spectra where then analysed via the Sequest HT Software created with Proteome Discoverer (version 1.4, Thermo Scientific) using the SwissProt *Mus musculus* database, containing 16,620 protein and Dystrophin 31aa Cter tail sequence (HNVGSLFHMADDLGRAMESLVSVMTDEEGAE). Carbamidomethylation of cysteines, oxidation of methionine, protein amino-terminal acetylation were set as variable modifications for all Sequest searches. Specificity of trypsin digestion was set and two missed cleavage site were allowed. The mass tolerances in MS and MS/MS were set to 5 p.p.m. and 0.5 Da, respectively. false discovery rate was set to 1% at the peptide level for all the searches.

### Statistical analysis

For statistical analysis, either Student's *t*-test or one-way analyses of variance were used as appropriate using GraphPad Prism software (Version 6, GraphPad Software Inc.).

## Additional information

**How to cite this article:** Rau, F. *et al*. Abnormal splicing switch of *DMD's* penultimate exon compromises muscle fibre maintenance in myotonic dystrophy. *Nat. Commun.* 6:7205 doi: 10.1038/ncomms8205 (2015).

## Supplementary Material

Supplementary Figures and Table Supplementary Figures 1-5, Supplementary Table 1

Supplementary Movie 1 Touch-evoked escape test of control embryos (1 image/0.2 sec).

Supplementary Movie 2 Touch-evoked escape test of moderate (0.1 mM AMO) *dmd* Δ78 morphants (1 image/0.2 sec).

Supplementary Movie 3 Touch-evoked escape test of severe (0.3 mM AMO) *dmd* Δ78 morphants (1 image/0.2 sec).
