# Supplementary material for: Abnormal splicing switch of DMD's penultimate exon compromises muscle fibre maintenance in myotonic dystrophy
Source: Nat Commun. 2015 May 28;6:7205. doi: 10.1038/ncomms8205 (PMC4458869; doi:10.1038/ncomms8205)
Supplement: Supplementary Figures and Table — Supplementary Figures 1-5, Supplementary Table 1 [file ncomms8205-s1.pdf]

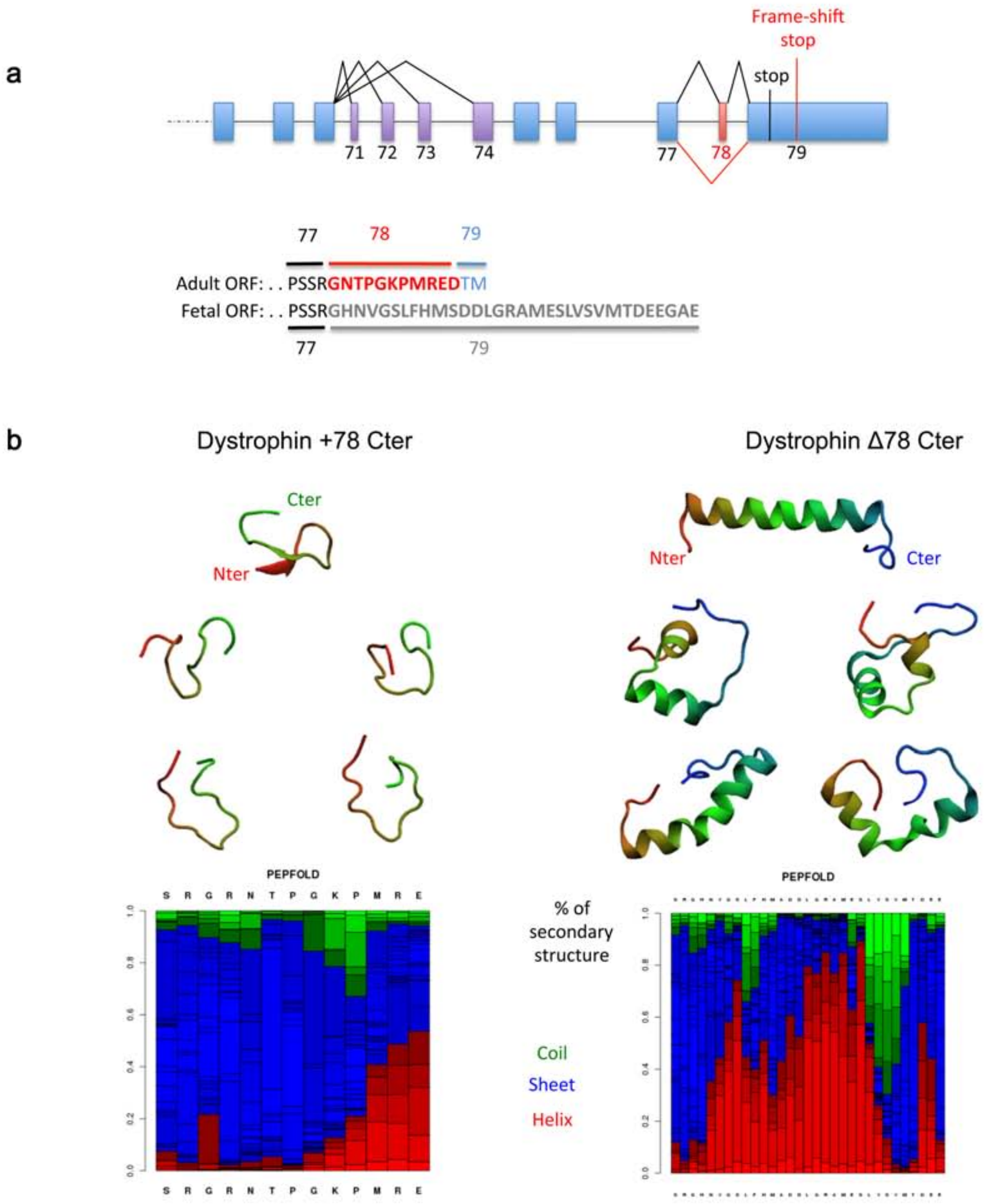

c

	Dystrophin +78 Cter				Dystrophin Δ78 Cter			
	polar	non-polar	total	% non-polar	polar	non-polar	total	% non-polar
model1	941,4	177,7	1119,1	15,9	1705,0	786,7	2491,7	31,6
model2	984,4	194,1	1178,5	16,5	1427,7	821,1	2248,8	36,5
model3	957,3	208,5	1165,9	17,9	1423,3	649,9	2073,3	31,3
model4	1050,0	187,9	1237,9	15,2	1443,1	746,6	2189,7	34,1
model5	1003,8	195,1	1198,9	16,3	1223,8	704,9	1928,7	36,5
average	987,4	192,7	1180,0	16,3	1444,6	741,9	2186,4	34,0
std	42,5	11,2	43,6	1,0	171,2	67,3	209,9	2,5

Supplementary Figure 1: a) Schematic representation of *DMD* exon 78 splicing and C-ter tail amino acid sequence in absence or presence of exon 78. **b)** Five models provided by PEP-fold program for the structure of dystrophin +78 and dystrophin Δ78 Cter tails. **c)** Molecular surface hydrophobicity of all PEP-fold models.

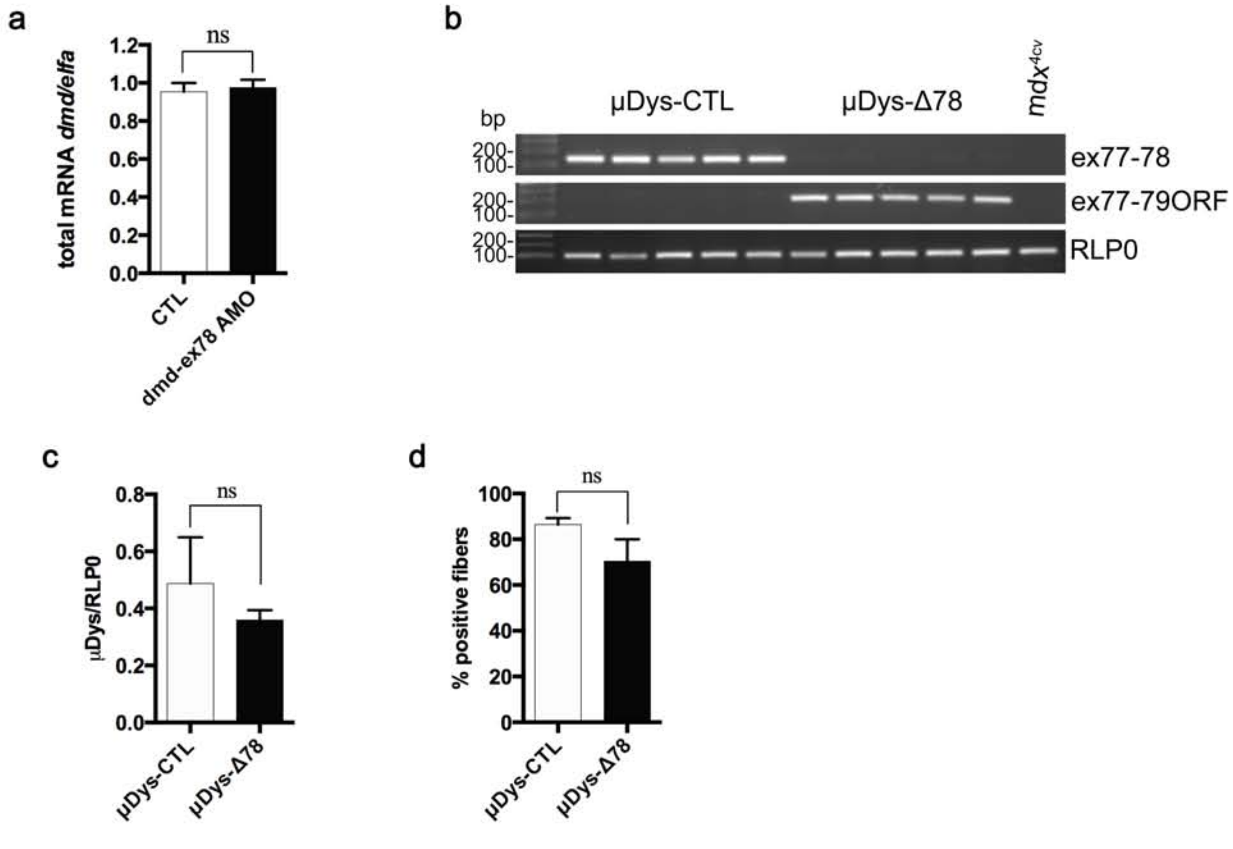

Supplementary Figure 2: a) qRT-PCR analysis of *dmd* mRNA levels in control embryos and *dmd*-exon 78 morphants at 48 hpf (from 3 independent experiments). **b)** RT-PCR analysis of µDys-CTL and µDys-∆ex78 mRNA levels in TA injected muscles (n=5). **c)** qRT-PCR analysis of µDys-CTL and µDys-∆ex78 mRNA level in TA injected muscles (n=8). **d)** Percentage of dystrophin positive fibers in µDys-CTL and µDys-∆ex78 injected muscles (n=5). Bars indicate s.e.m and "ns" indicates not significant; Student *t-test*.

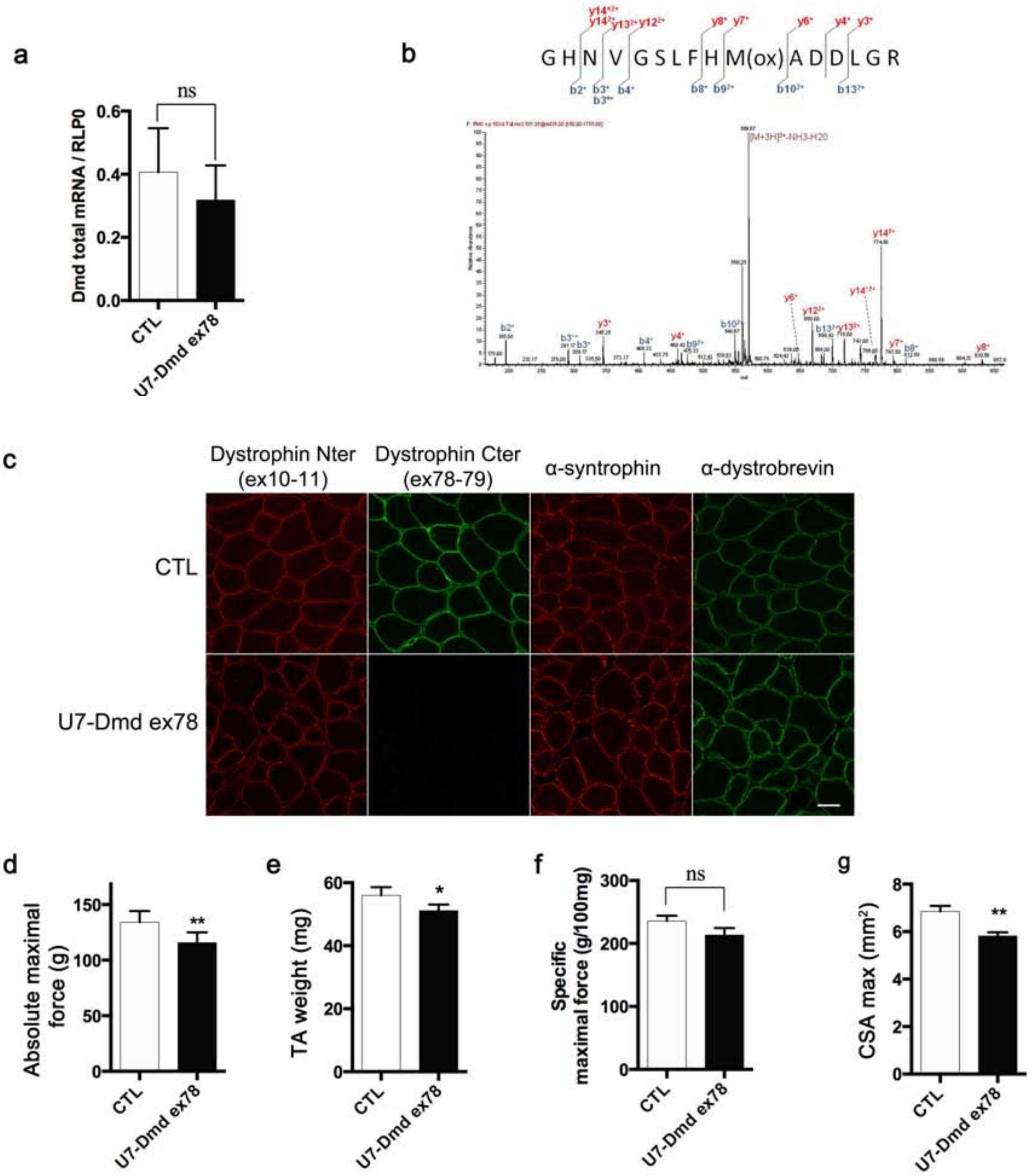

Supplementary Figure 3: a) qRT-PCR analysis of total *Dmd* mRNA level in U7-Dmd exon 78 injected muscles compared to saline-injected muscles, 6 months after injection (n=10). **b)** Tandem mass Spectrum (MS/MS) of the precursor ion at m/z 581.25 determining oxidized peptide ^{3665}GHNVGSLFHMoxADDLGR^{3680}. The fragmentation Spectrum shown is a trypsin-derived peptide. The inset show the peptide sequence and the observed ions obtained in the MS/MS Spectrum labeled to show singly and doubly charged *b* and *y* ions, as well as ion corresponding to neutral losses of water (circles) and NH3 (asteriks); M, parent ion mass. **c)** Dystrophin N-terminal domain (ex10-11; Manex1011B antibody), Dystrophin C-terminus domain (ex78-79; Dys2 antibody), α-syntrophin and α-dystrobrevin immunostaining of TA muscles injected with U7-*Dmd* exon 78 compared to saline-injected TA muscles (scale bar, 50µm). **d)** Absolute maximal muscle force (P0) of U7-*Dmd* exon 78 TA injected muscles compared to saline-injected muscles (n=10). **e)** TA muscles weight of U7-*Dmd* exon 78 injected muscles compared to saline-injected muscles (n=10). **f)** Specific maximal force (sP0) of U7-*Dmd* exon 78 TA injected muscles compared to saline-injected muscles (n=10). **g)** Maximal cross-section area of U7-*Dmd* exon 78-injected TA muscles compared to saline injected-TA muscles (n=10). Bars indicate s.e.m and “ns” indicates not significant; * indicates $p<0.05$; ** indicates $p<0.01$; paired *t-test*.

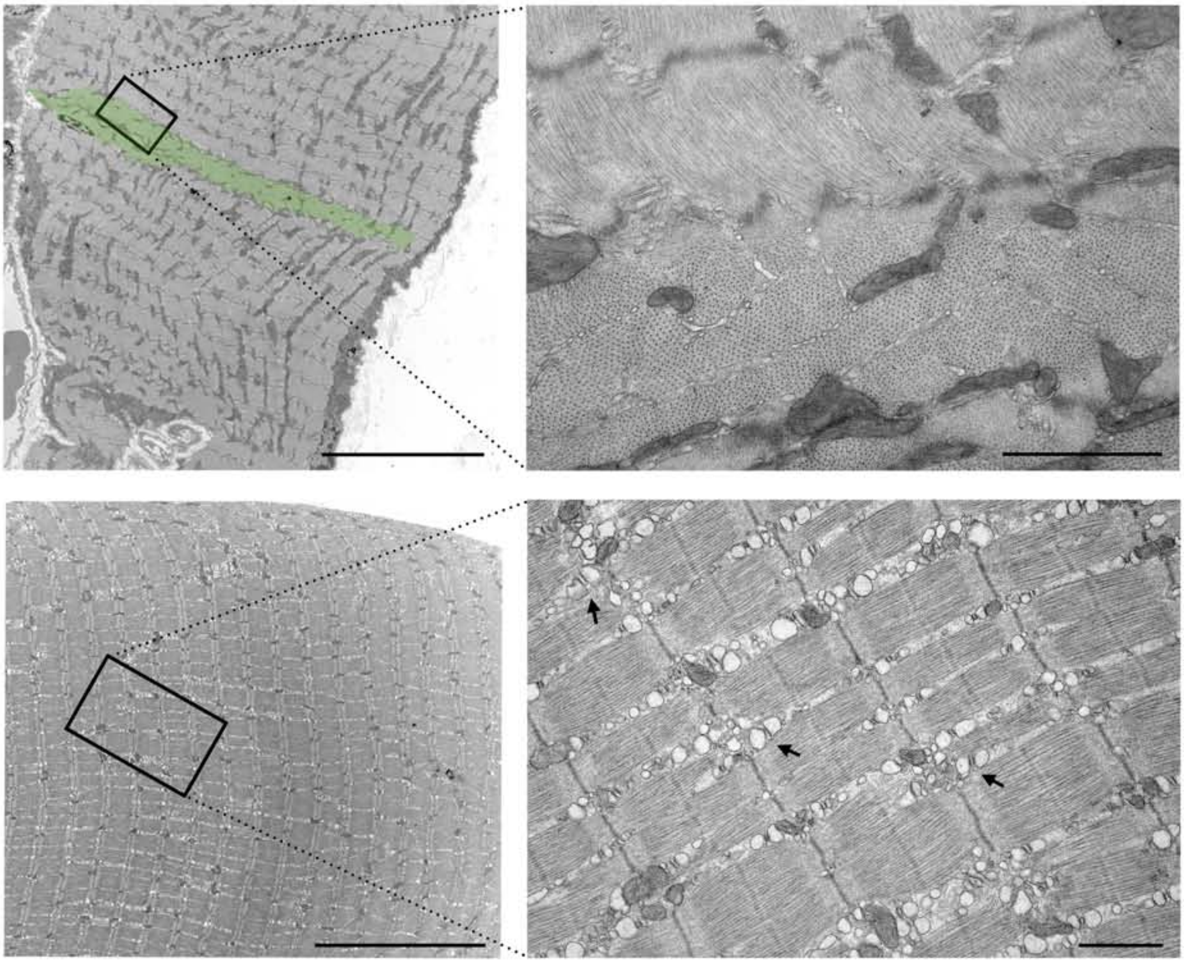

Supplementary Figure 4: Upper panel: Another example of disorientated myofibrils (pseudo-colored in green) within an oxidative myofiber: a band of myofibrils running at right angle to the fiber major direction traverse the whole fiber. **Lower panel:** Dilated sarcoplasmic reticulum in a fast twitch fiber. Arrows point triads with swollen reticular terminal cisternae Scale bar, 10μm in the right panels and scale bar, 1μm in the left panels.

**Primers for splicing**		
	**primer FW**	**primer Rev**
human *DMD* ex78	TTAGAGGAGGTGATGGAGCA	GATACTAAGGACTCCATCGC
mouse *Dmd* ex 78	TGGTTGGCAGTCAAACTTCA	TCATCTGCCATGTGGAAAAG
zebrafish *dmd* ex78	CCCAGGATGCAAGCACTGGATTAG	TTACATGAACCAGCGACTCC
**Primers for RT-PCR**		
	**primer FW**	**primer Rev**
mouse μDys-CTL	CTCTCAGACCAGCGAGAGCA	CTCCCGCATGGGCTTGCCGG
mouse μDys-Δ78	CTCTCAGACCAGCGAGAGCA	ACTCCATCGCTCTGCCCAAA
**Primers for qRT-PCR**		
	**primer FW**	**primer Rev**
zebrafish *elfa*	CTTCTCAGGCTGACTGTGC	CCGCTAGCATTACCCTCC
zebrafish *dmd*	GGAGCTGACGTCTCACCAG	TGCTCTGTCGCTCCATACTG
mouse *Rlp0*	GAGGACCTCACTGAGATTCGG	TTCTGAGCTGGCACAGTGAC
mouse *Dmd*	TGGATCTGACATCTCATCAAGGAC	CCATGCTAGCTACCCTGAGAC
mouse *μDys*	AGGCAGAGCACCAGAAACTACC	CTGGCACTTGGCGATGTTGAAG
mouse *Myh1*	GCGAGGTTCACACCAAAATCA	TGGTCACTTTCCTGCTTTGGA
mouse *Myh2*	AAGCGAAGAGTAAGGCTGTC	GTGATTGCTTGCAAAGGAAC
mouse *Myh4*	ACAAGCTGCGGGTGAAGAGC	CAGGACAGTGACAAAGAACG

Supplementary Table S1: Primer sequences used for RT-PCR and qRT-PCR analysis.

Figure 1c

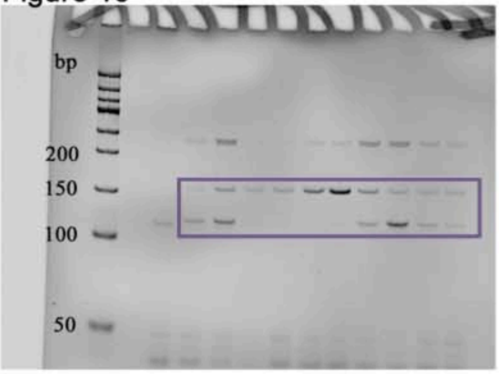

Figure 1e (left panel)

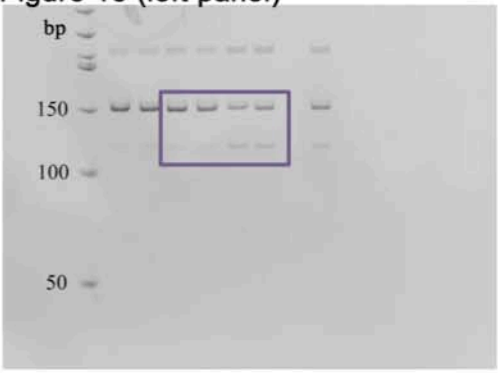

Figure 1e (right panel)

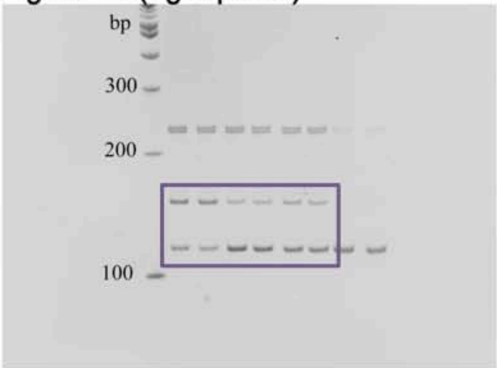

Figure 1f

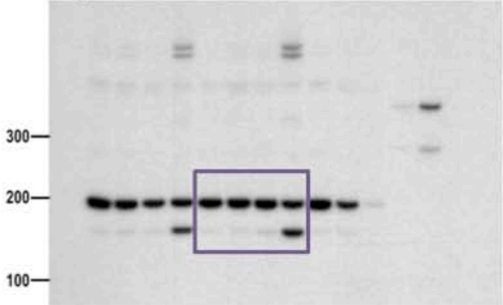

Figure 2a

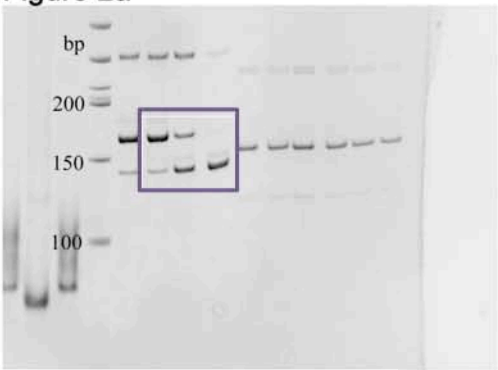

Figure 4a

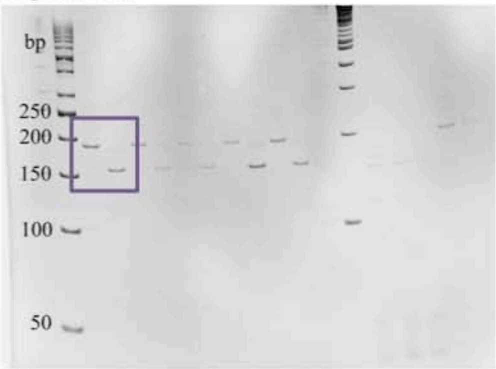

Supplementary Figure 2b

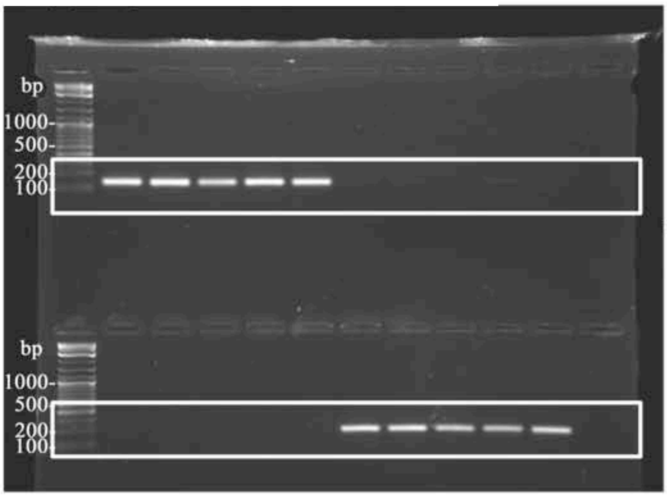

Supplementary Figure 2b

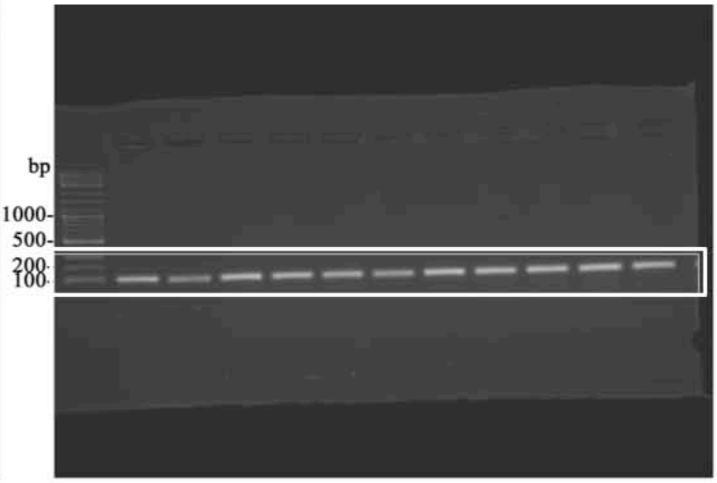

Supplementary Figure 5: Uncropped PCR gels
